# Impact of the Atlantic Multidecadal Oscillation on the Pacific North Equatorial Current bifurcation

**DOI:** 10.1038/s41598-019-38479-w

**Published:** 2019-02-15

**Authors:** Chau-Ron Wu, Yong-Fu Lin, Bo Qiu

**Affiliations:** 1 Department of Earth Sciences, National Taiwan Normal University, Taipei, Taiwan; 2 Department of Oceanography, University of Hawaii at Manoa, Honolulu, Hawaii USA

## Abstract

Variability in surface currents is mainly induced by the time-varying wind forcing. Recent studies have revealed robust pronounced changes in the atmospheric circulation over the Pacific came with the Pacific Decadal Oscillation (PDO). However, the PDO is a climate index based on sea surface temperature (SST) variations, and may not be appropriately considered as a climate forcing. Here, we suggest the Atlantic SST variability is the ultimate forcing leading to changes in the atmospheric circulation and surface winds over the North Pacific. Anomalous warm North Atlantic and cold South Atlantic leads to weak Hadley cell in the Northern Hemisphere, resulting in a northward displacement of the ITCZ as well as a positive wind stress curl anomaly in the Pacific subtropical region, which would reduce the North Pacific subtropical gyre (NPSG). Associated with reduced sea surface height in the subtropics by the weakened NPSG, the North Equatorial Current (NEC) is weakened based on geostrophy. Changes in basin-scale winds further result in the southward migration of the tropical gyre and consequential downstream ocean circulation.

## Introduction

**Figure 1:**
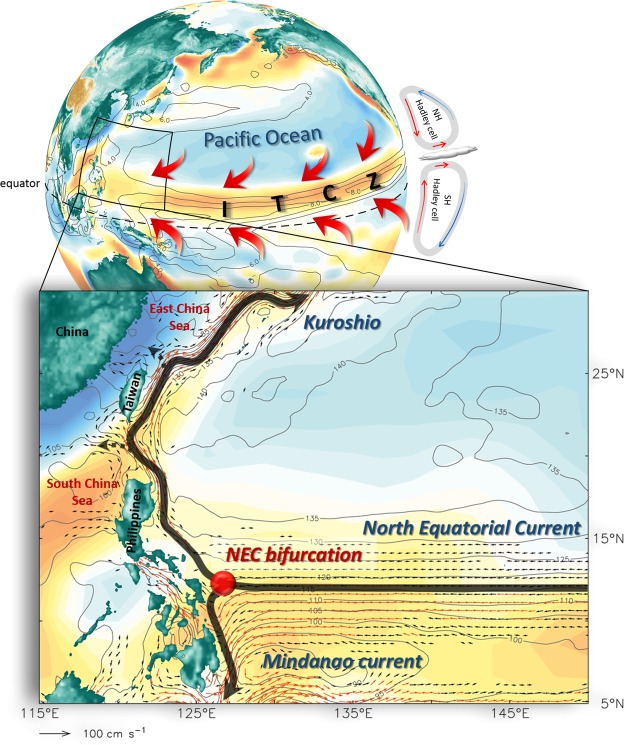
Pacific Ocean wind field and boundary currents. All colors denote annual mean wind stress curl, and warm (cold) color indicates the anticyclonic (cyclonic) wind field. Contours are annual mean precipitation (unit in mm day^−1^, top) from GPCP products provided by the NOAA/OAR/ESRL PSD from their website at https://www.esrl.noaa.gov/psd/, and SSH (unit in cm, bottom) from AVISO delayed-time maps of Absolute Dynamic Topography (DT-MADT) products processed by Ssalto/Duacs and distributed by AVISO+ with support from CNES (https://www.aviso.altimetry.fr/duacs/), respectively. Red arrows (top) denote northeast and southeast trade winds. Arrows (bottom) are mean surface ocean currents in the northwestern Pacific based on AVISO DT-MADT geostrophic velocities products, which altimeter products were produced by Ssalto/Duacs and distributed by Aviso+, with support from CNES (https://www.aviso.altimetry.fr/duacs/). Black and red arrows indicate current velocities with speeds of 10–20 cm s^−1^ and >20 cm s^−1^, respectively.

In the western North Pacific Ocean, the westward-flowing North Equatorial Current (NEC) bifurcates as it encounters the Philippine coast (Fig. 1). At the crossroads of the tropical and subtropical circulations, the bifurcated NEC provides important pathways for heat, mass, and salt transport exchanges between the low-latitude and mid-latitude North Pacific Ocean^1^. The poleward-flowing bifurcated branch is the Kuroshio which flows northward and northeastward off the Luzon, Taiwan, and Japan, ultimately recirculating eastward as the Kuroshio Extension. The Kuroshio modulates the climate by transporting excess heat from tropics poleward^2,3^. It also affects regional climate, typhoon development^4^, fishery economy^5,6^ as well as ocean circulation and hydrography in surrounding marginal seas such as the East China Sea (ECS) and South China Sea (SCS)^7,8^.

Several studies based on model outputs and satellite altimeter measurements revealed a similar seasonality of the NEC bifurcation latitude (NECBL), migrating southward in summer and northward during wintertime^9,10^. Beyond the seasonal time scale, the NECBL also varies on an interannual timescale^11^. Until recently, most studies attributed the interannual variability of the NECBL to the El Niño–Southern Oscillation (ENSO), which made a northward shift during El Niño and a southward shift during La Niña^11^. Lately, Wu^8^ demonstrates that the impact of ENSO on the NECBL is not stationary, and it depends on the phase of the Pacific Decadal Oscillation (PDO). The NECBL variations only show a close correspondence with the ENSO during the cold PDO phase.

In the tropical Pacific, variability in surface currents is mainly induced by the time-varying wind forcing. For example, Qiu and Lukas^11^ demonstrated that the NEC is controlled by basin-wide wind stress curl anomaly (WSCA) and wind fluctuations would trigger a meridional shift in the NECBL. Qiu and Chen^10^ further noted that the exact location of the NECBL is largely determined by wind forcing in a band defined by 12°N–14°N and 140°E–170°E. Fundamentally, the meridional migration of the NEC is closely related to that of tropical atmospheric circulation. Several recent studies pointed out that pronounced changes in the atmospheric circulation and surface winds over the Pacific came with the PDO. For example, a canonical negative PDO normally contains enhanced trade winds^12–14^ and weakened westerlies^15^. However, the PDO is a climate index based on the North Pacific sea surface temperature (SST) variations^16^, and may not be appropriately considered as a climate forcing. In this study, the ultimate forcing leading to changes in the atmospheric circulation and surface winds over the North Pacific is examined, and the multidecadal variability of the Atlantic Multidecadal Oscillation (AMO) is proposed to the likely candidate. The AMO is the dominant mode of SST variability in the Atlantic Ocean. Previous studies indicate that the AMO behaves as a mixture of many drivers, such as Atlantic meridional overturning circulation (AMOC)^17–20^, external radiative forcing^21,22^, and atmospheric low-frequency variability^23,24^.

Atmospheric circulation in the tropics is associated with the Walker circulation zonally across ocean basins. Tropical SST variations generally vary in response to changes in the Walker circulation, which are forced externally through seasonal shift of the Sun or resulted from coupled atmosphere-ocean feedback. Recent studies suggest that the Walker circulation plays a significant part in the transbasin connections between the tropical Atlantic and Pacific Oceans under conditions of climate change^25–33^. Walker circulation aside, Hadley cell, a global scale meridional circulation with an ascending branch over equatorial areas and sinking over the subtropical latitudes (around 30° of latitude) in both the Southern and Northern Hemispheres (Fig. 1), is also an important factor affecting atmospheric circulation in the tropics.

Precipitation in the tropics exists largely within a narrow zonal band, known as the Inter-tropical Convergence Zone (ITCZ), which is also the area where the northeast and southeast trade winds converge (Fig. 1). Previous studies demonstrated that the intensity of Hadley cell has a significant impact on the meridional migration of the ITCZ (ref.^34^). In boreal summer, the ITCZ is displaced toward the warmer Northern Hemisphere where the Hadley cell is weakened due to the meridional temperature gradient of both ocean and atmosphere is smaller. On the contrary, the ITCZ generally migrates to the warmer Southern Hemisphere in boreal winter^35^. Consequently, the location of the ITCZ could be served as a good proxy for the intensity of Hadley cell. Changes in basin-scale wind field induced by the Hadley circulation result in not only the ITCZ variability but the tropical ocean circulation, especially the NEC migration. Meridional migrations of both the NEC and ITCZ are thus manifestations of the time-varying near-surface tropical atmospheric circulation.

### Climate variability responsible for the NECBL fluctuation

**Figure 2:**
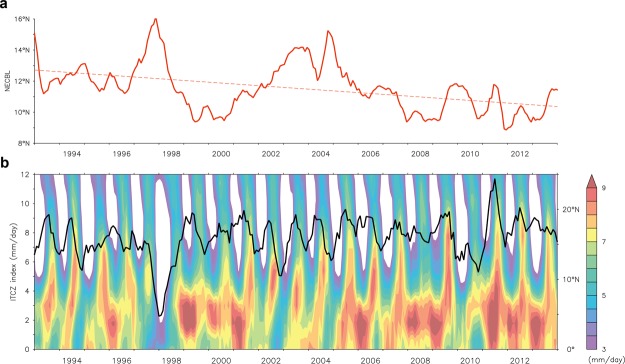
Time series of monthly NECBL and the ITCZ index. Time series of (**a**) monthly NECBL (NEC bifurcation latitude). Dashed line denotes the linear trend of NECBL. (**b**) Monthly precipitation (averaged over 110°–160°E, colors) and the ITCZ index (black curve) with 5-month running mean.

Figure 2a shows time series of the NECBL estimated from the AVISO altimeter data during 1993–2013. The time series reveals a significant year-to-year variation, that has a range from ~10°N in 1996 to ~16°N during 1997/98 when it was at the mature phase of the strongest El Niño event in the last century. Upon further inspection, it is shown that changes in the NECBL seem to be more closely relating to the PDO than ENSO during 1993–2013 (r = 0.87, P < 0.001; see Fig. S1), compatible with the previous study by Wu^8^. Interannual timescale variability aside, the NECBL likely has a long-term trend (dashed line in Fig. 2a), and has progressively shifted to a southern latitude over the past 21 years.

The ITCZ is the main rainfall region, and precipitation anomalies averaged over the domain between 0–10°N and 130–160°E serve as a good indicator for the location of the ITCZ in the northwestern tropical Pacific (denoted as the ITCZ index hereafter) as demonstrated by Lin *et al*.^36^. Figure 2b shows monthly precipitation averaged over 110–160°E and the ITCZ index during the period from 1993 to 2013. Beyond the seasonal time scale, precipitation and ITCZ also exhibit a significant interannual variation. The ITCZ index generally migrates between 12°N and 19°N except that a significant southward movement (to ~3°N) appears during the strongest El Niño event in 1997/98. It is virtually out of phase with the NECBL shown in Fig. 2a. The two time series are consistent, with a correlation coefficient of −0.52, which is higher than the 99% significance level.

### Transbasin influence of the Atlantic Ocean on the Pacific climate

The AMO appears to modify climate conditions over many regions, and contributes to multidecadal fluctuations of the global mean surface temperature^37,38^, especially in the Northern Hemisphere. Not only confined to Atlantic and North American climate, many prominent multidecadal variabilities in the Pacific climate have been demonstrated to be related to the AMO, such as the intensity of Southeast and East Asian summer monsoons^39^, and modulation of the ENSO variability^40–43^. Those studies have shed important light on Pacific climate variabilities in response to the imposed forcing in the Atlantic. Although the description in the study, impact of the North Atlantic variability on the Pacific, is similar to that in Rodríguez-Fonseca *et al*.^41^, Ding *et al*.^42^ or Polo *et al*.^43^, we expand its consequent influence on ocean circulation, such as the NECBL and North Pacific subtropical gyre (NPSG) in response to Hadley cell changes.

**Figure 3:**
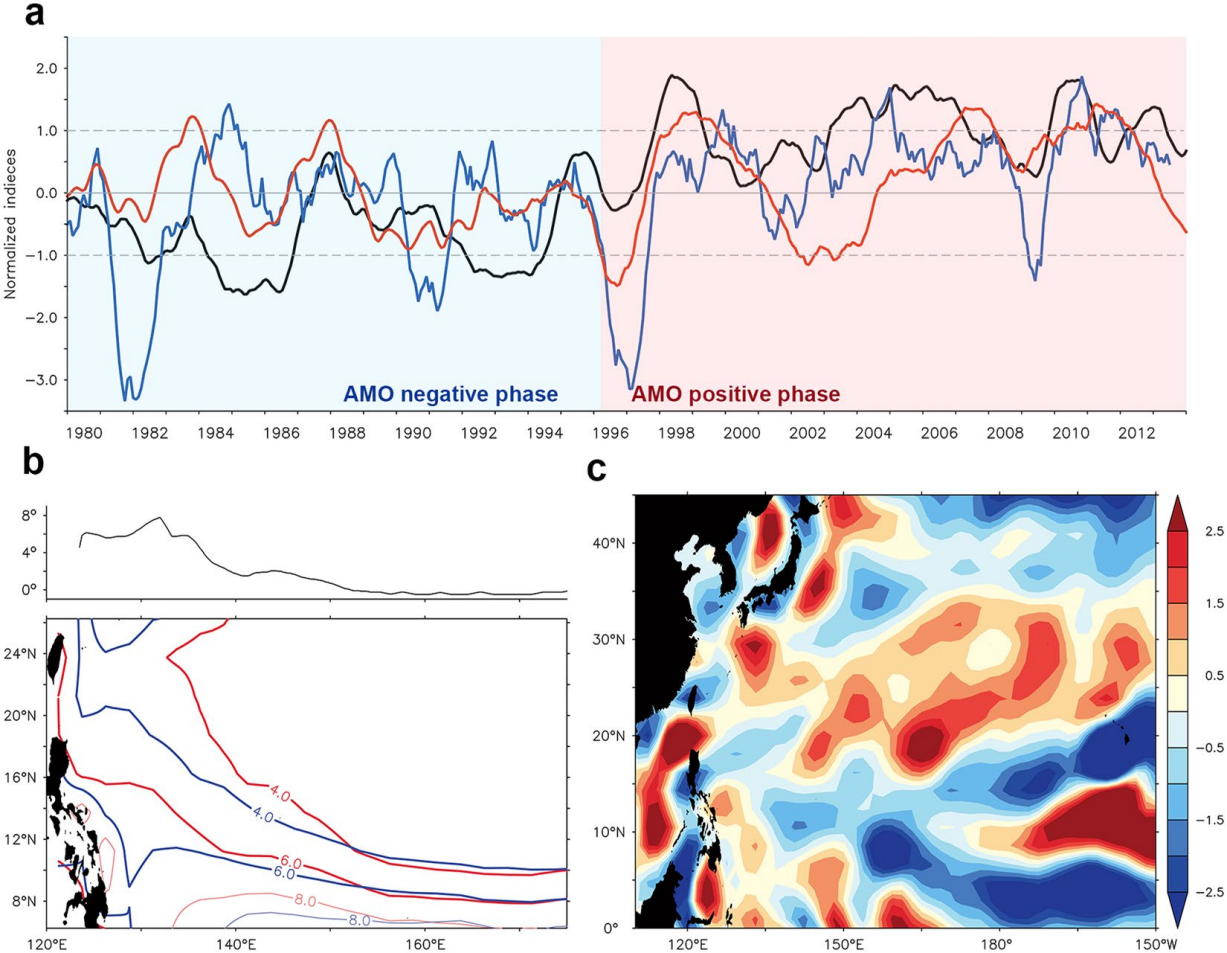
Impact of the AMO on the North Pacific. (**a**) Monthly time series of three indices with 15-month running mean and normalized. Black curve is the AMO index, red curve is the NECBL (multiplied by -1), and blue curve is the ITCZ index. The ITCZ index and NECBL are shifted ahead of 13 months. Gray dashed line shows one standard deviation of the mean AMO index. Blue (red) shading denotes the negative (positive) AMO phase. (**b**) Difference of the ITCZ latitude (northern boundary of major tropical convection, >4 mm day^−1^) between the positive and negative AMO phase (top), and comparison of precipitation (unit in mm day^−1^, bottom) composited for the positive phase (red) and negative AMO phase (blue) during 1980–2013. (**c**) The WSCA difference (unit in 10^−8^ N m^−3^) between the positive and negative AMO phase during 1980–2013. The WSCA and precipitation composites are performed over episodes when the positive (negative) AMO index is larger (smaller) than one standard deviation.

Figure 3a shows the time series of normalized AMO index (black curve), together with normalized ITCZ index (blue curve) and NECBL (red curve) during 1980–2013. A 15-month running mean is applied to all the three time series. For clarity, both the ITCZ index and NECBL are shifted ahead of 13 months because the AMO leads the two by 13 months with correlation coefficients higher than the 90% significance level (Table S1). The close correlation suggests the impact of the Atlantic SST variability on Pacific climate, and that agrees with recent findings^44^. A diagnosis based on precipitation patterns is performed to illustrate the transbasin influence of the Atlantic Ocean on Pacific climate and variability.

Figure 3b shows a comparison of the precipitation composite for the positive and negative AMO phase during 1980–2013. The composite is performed over the episodes when the positive (negative) AMO index is larger (smaller) than one standard deviation, which filters out ENSO signals to a certain extent. Geographically, the rainfall zone (or the ITCZ) in the positive phase is farther north compared with that in the negative phase, particularly in the northwestern tropical Pacific. Beyond the seasonal timescale which is mainly sun-driven, the time-varying ITCZ could be understood by a global energetic framework proposed by Frierson *et al*.^45^. By both observations and model simulations, Frierson *et al*.^45^ demonstrated that the meridional overturning circulation contributes significantly to the hemispheric asymmetry in tropical rainfall by transporting heat from the Southern Hemisphere to the Northern Hemisphere, and thereby pushing the tropical rain band north.

The positive AMO phase suggests an anomalous warm North Atlantic and cold South Atlantic, resulting in ocean releases more heat in the Northern Hemisphere than Southern Hemisphere, which leads to a weakened Hadley cell in the Northern Hemisphere but a strengthened Hadley cell in the Southern Hemisphere. Changes in the Hadley cell resemble the observations which is evident not only in the Atlantic but in the Pacific (Fig. S2). Applying the energetic framework^45^, the ITCZ in the positive AMO phase should be farther north compared with that in the negative phase as shown in Fig. 3b. Associated with the weakened Hadley cell, eddy transport is reduced in the midlatitudes of the Northern Hemisphere^45^, which would result in weakened westerlies and positive WSCA in the subtropical Pacific. Figure 3c shows the WSCA difference between the positive and negative AMO phase during 1980–2013. A positive WSCA in the subtropical region indicates a weakened NPSG. Alternatively, the tropical Atlantic warming associated with the positive AMO phase leads to changes in the Pacific Walker circulation and induces anomalous descending motion over the central tropical Pacific^27,30^. The descending motion is capable of exciting poleward-propagating Rossby waves, which may also contribute to the weakened NPSG (ref.^33^).

A correlation map illustrating the relationship between the AMO and WSCA in the North Pacific is shown in Fig. S3. The AMO and WSCA are closely related, with the AMO leads WSCA by 13 months. The correlation map exhibits that there is a positive (negative) WSCA in the North Pacific subtropical region during the positive (negative) AMO phase. The wind-induced sea surface height (SSH) is reduced in the subtropics, which leads a weaker meridional pressure gradient, weakening the NEC. The weakened tendency of the NEC is also confirmed by the tidal-gauge-based transport (figure not shown). Furthermore, Hadley cell in the Southern Hemisphere is intensified, and so is its ascending branch. The strengthening southeast trade winds induce a broad-scale negative Ekman flux divergence in the zonal band of 10°N–15°N and 140°E–170°E (Fig. 3c), which causes a southward migration of the tropical gyre and consequently the southward migration of the NEC.

**Figure 4:**
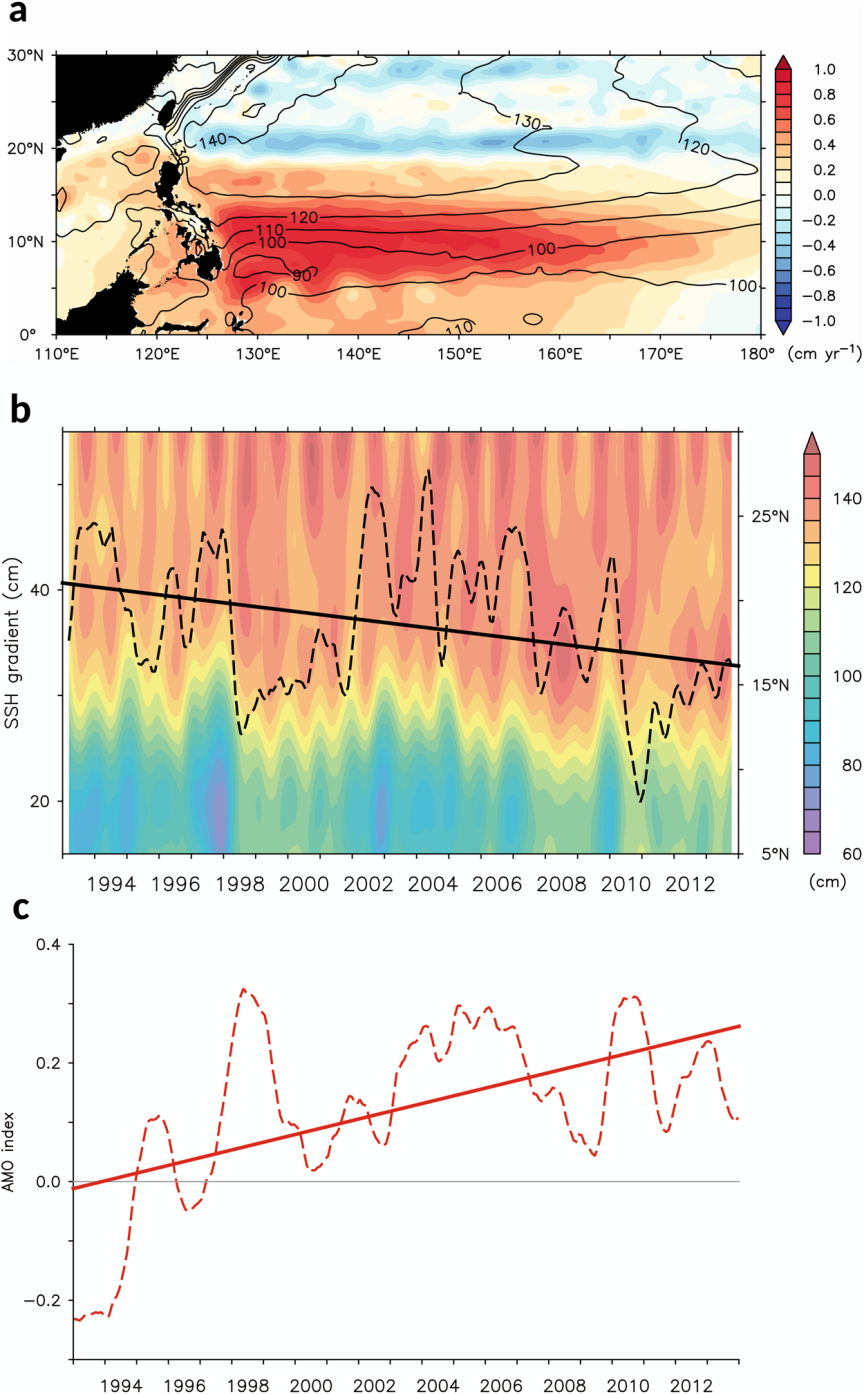
Sea level variability during the past two decades. (**a**) Annual mean SSH (contours) and linear trend of SSH (colors) in the North Pacific during 1993–2013. Linear trend of global SSH (~0.29 cm yr^−1^) was removed. (**b**) Monthly time series of SSH averaged over the 130°–150°E with 5-month running mean in major latitudes of interest (5–30°N) during 1993–2013. Black dashed line is meridional SSH gradient (18–14°N minus 6–10°N), and black solid line is linear trend of the SSH gradient. (**c**) Monthly time series of the AMO index with 15-month running mean (dashed line). Solid line is linear trend of the AMO index.

The SSH variability is further shown in Fig. 4a, depicting the annual mean SSH from AVISO (contours) and the linear trend of SSH (colors) in the North Pacific during 1993–2013. Linear trend of global mean SSH (~0.29 cm yr^−1^) was removed. Geographically, the mean SSH field reveals that the NEC bifurcates at a mean latitude of 12°N off the Philippines. This mean bifurcation latitude for the surface NEC agrees favorably with the values inferred previously based on historical hydrographic and surface drifter data^2,46,47^. Furthermore, the dominance of negative SSH trend shows up to the north of 20°N in the subtropics, which is resulted from the positive WSCA tendency during the 1993–2013. On the other hand, positive SSH trend shows up as patches south of 20°N with a vigorous increase south of 14°N in the Pacific warm pool. The SSH gradient across the westward-flowing NEC tends to be smaller accordingly. Based on geostrophy, the NEC is weakened due to a weaker meridional pressure gradient trend.

The detailed tendency is illustrated in Fig. 4b, which shows the SSH variability averaged over the 130°–150°E, together with its meridional gradient (18–14°N minus 6–10°N, black curve) in latitudes of interest (5–30°N) during 1993–2013. Geographically, the raised SSH tends to migrate southward, indicating a weakened tendency of the meridional gradient during 1993–2013. The southward raised SSH also implies the southward migration of the tropical gyre and consequently the southward migrating trend of the NEC, which agrees with the previous studies^48,49^. Furthermore, this tendency is in response to the recent North Atlantic warming (or the positive trend of AMO shown in Fig. 4c). The southward migration of the NECBL will lead to enhance the Kuroshio transport east of Luzon, and the Kuroshio has tended to bypass the Luzon Strait without significant westward encroachment^49,50^.

### Conclusion and Discussion

**Figure 5:**
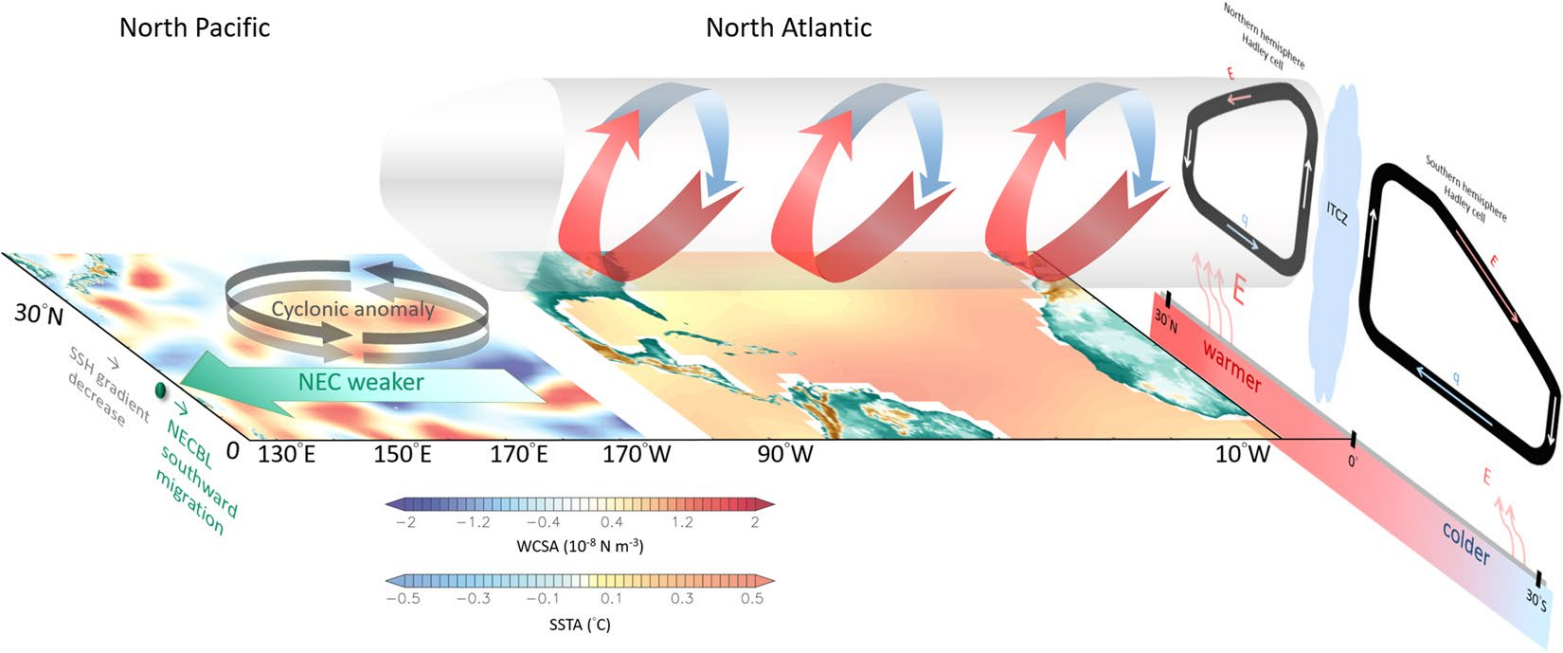
Diagram of the linkage between the Atlantic and North Pacific. Anomalous warm North Atlantic and cold South Atlantic leads to weak Hadley cell in the Northern Hemisphere but strong Hadley cell in the Southern Hemisphere, resulting in a northward displacement of the ITCZ not only in the Atlantic but also in the Pacific. The weakened Hadley cell leads to a positive WSCA in the Pacific subtropical region, which would result in a weakened NPSG. The NEC is weakened and southward migrating because the meridional pressure gradient across it is reduced, which is associated with reduced SSH in the subtropics by the weakened NPSG.

The current study presents the transbasin influence of the Atlantic climate variability on the Pacific NECBL fluctuation while Fig. 5 shows a schematic representation of the sequence among atmospheric/oceanic variabilities in the Atlantic and Pacific Oceans. In the mid-1990s, the North Atlantic undergoes a remarkable warming, which leads to weak Hadley cell in the Northern Hemisphere but strong Hadley cell in the Southern Hemisphere, consequently resulting in a northward displacement of the ITCZ not only in the Atlantic but also in the Pacific. A weakened Hadley cell in the Northern Hemisphere leads to a positive WSCA in the Pacific subtropical region (see also Fig. 3c), which would result in a weakened NPSG. The NEC is weakened and southward migrating because the meridional pressure gradient across it is reduced, which is associated with reduced SSH in the subtropics by the weakened NPSG.

Hadley cell variability aside, Walker circulation also revealed its important role on the Atlantic-Pacific connection. Several recent studies pointed out that the recent North Atlantic warming impacted the Pacific through a modification of the Walker circulation^34–44^. The Pacific Walker circulation changes evoked anomalous descending motion over the central tropical Pacific^27,30,33^. The deep atmospheric convection anomalies happening could generate Tropical-Extratropical Rossby waves propagating northward, which would also impact the NPSG (ref.^33^) and consequential NEC intensity and its migration.

## Methods

Ocean surface velocities and sea level data are adopted from Satellite Oceanographic data from AVISO products processed by Ssalto/Duacs and distributed by AVISO+ with support from the National Centre for Space Studies (CNES) (https://www.aviso.altimetry.fr/duacs/). The AVISO products (AVISO version DTMADT and DT-MSLA, two sat merged of Ssalto/Duacs) include surface geostrophic velocities, Absolute Dynamic Topography (ADT), and sea level anomaly that are sampled daily with a spatial resolution of 0.25°. The NEC bifurcation latitude used the wind-forced Rossby wave model (1979–1992) is calculated from monthly AVISO data (after 1992)^10^.

Precipitation data are from the Global Precipitation Climatology Project Version 2.2 (GPCP V2.2, http://www.esrl.noaa.gov/psd/data/gridded/data.gpcp.html) monthly precipitation product, which is based on a blend of satellite and *in-situ* measurements since 1979 on global grids (2.5° × 2.5°)^51^. Wind stress data are from National Centers for Environmental Prediction/Department of Energy (NCEP/DOE) AMIP Reanalysis2 (https://www.esrl.noaa.gov/psd/data/gridded/data.ncep.reanalysis2.html) with a 2.5° latitude by 2.5° longitude resolution. The NCEP/DOE AMIP Reanalysis-2 (R-2) is an improved version of the NCEP/NCAR Reanalysis-1 (R-1) that fixed errors and updated parameterizations of physical processes.

The AMO index is calculated as the detrended SST anomalies averaged over the North Atlantic from the equator to the 70°N^52^.

### Statistical analyses

Lagged correlations have been made from low-pass-filtered time series. Significance level was calculated using a standard *t*-test. Auto-correlation is taken into account by adjusting the effective number of independent observations.

## Supplementary information

Supplementary Info
