# Supplementary material for: Impact of the Atlantic Multidecadal Oscillation on the Pacific North Equatorial Current bifurcation
Source: Sci Rep. 2019 Feb 15;9:2162. doi: 10.1038/s41598-019-38479-w (PMC6377656; doi:10.1038/s41598-019-38479-w)
Supplement: Supplementary file 1 — Supplementary Info [file 41598_2019_38479_MOESM1_ESM.doc]

**Supporting Online Material for:**

**Impact of the Atlantic Multidecadal Oscillation on the Pacific North Equatorial Current bifurcation**

Chau-Ron Wu^1^, Yong-Fu Lin^1^, and Bo Qiu^2^

^1^Department of Earth Sciences, National Taiwan Normal University, Taipei, Taiwan

^2^Department of Oceanography, University of Hawaii at Manoa, Honolulu, Hawaii

**1. Correlation between PDO and NECBL**

Figure S1 shows that changes in the NECBL are closely relating to the PDO during 1993-2013, with a correlation coefficient of 0.87, which is higher than the 99% significance level.

**2. Correlation between AMO and ITCZ (or Hadley circulation)**

Figure S2 demonstrates Hadley circulation changes in response to an AMO phase change during 1980-2013 based on NCEP-r2 dataset. It shows that weak Hadley cell in the Northern Hemisphere but strong Hadley cell in the Southern Hemisphere would result in a northward displacement of the ITCZ not only in the Atlantic but also in the Pacific.

Figure S3 shows a correlation map that illustrates the relationship between the AMO and wind stress curl anomalies (WSCA) in the North Pacific. Graphically, the AMO and WSCA are closely related, with the AMO leads the WSCA by 13 months. The correlation map exhibits that there is a positive (negative) WSCA in the North Pacific subtropical region during the positive (negative) AMO phase. Furthermore, Table S1 presents the correlations between indices of the AMO, ITCZ and NECBL.

**3. Numerical model experiments**

To better examine the transbasin influence of the Atlantic on Pacific, numerical model experiments were performed using the NCAR Community Atmospheric Model, version 3.0, (CAM3.0)^1^ with a T42 Eulerian spectral resolution (128 × 64 grid points) and 26 vertical levels. In this set of the experiments, the AGCM is coupled to a mixed layer slab ocean model (SOM) in other ocean basins. SST anomalies were prescribed in the North Atlantic (0–70°N) to represent the positive AMO phase in one experiment (i.e., AMO-positive experiment) and the negative AMO phase in the other experiment (i.e., AMO-negative experiment). The coupled model was integrated for 120 years for each of the two experiments, and model output from the last 100 years were used for the present analysis. Using only the last 40 years of the AGCM–SOM output gives very similar results. The simulated AMO responses were defined as the mean state differences between the AMO-positive and AMO-negative experiments.

Figure S4a shows SST differences between the positive and negative AMO phases of the modeling experiments. The North Atlantic SSTA pattern associated with the positive phase of AMO has a horseshoe-like shape, with two warming regions in the tropical and extratropical North Atlantic separated by a less-warm bands, resembling the pattern observed in regression analyses^2^. Figure S4b shows wind stress curl differences between the two experiments. Graphically, the positive WSCA in the subtropical region resemble those observed (see Figure 3c). This modeling result further confirms the argument that the AMO phase change is likely the cause for the positive WSCA in the subtropical Pacific region.


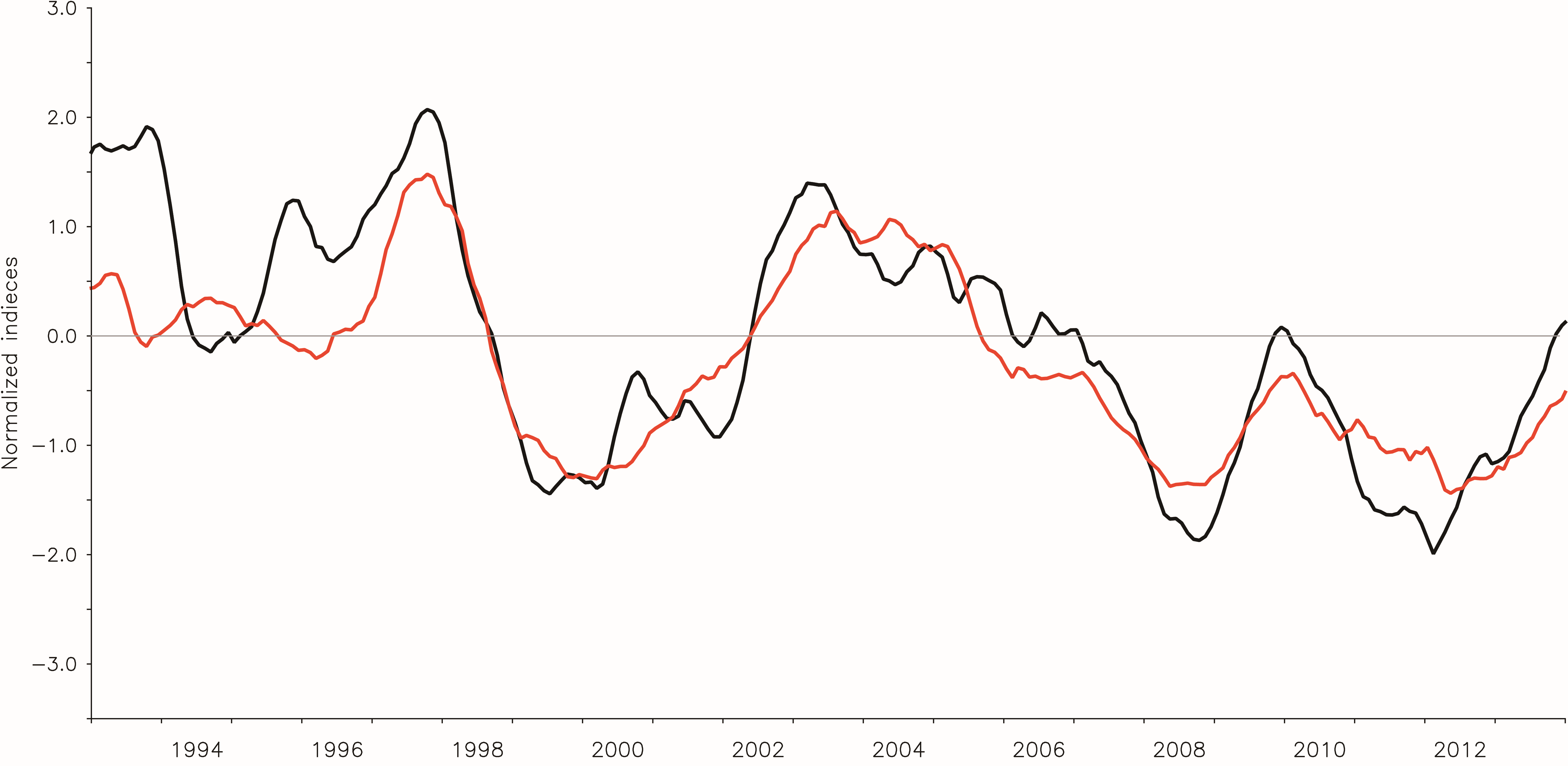


**Figure S1.** Monthly time series of the PDO index (black curve) and NECBL (red curve). A 15-month running mean was applied to both of normalized time series.


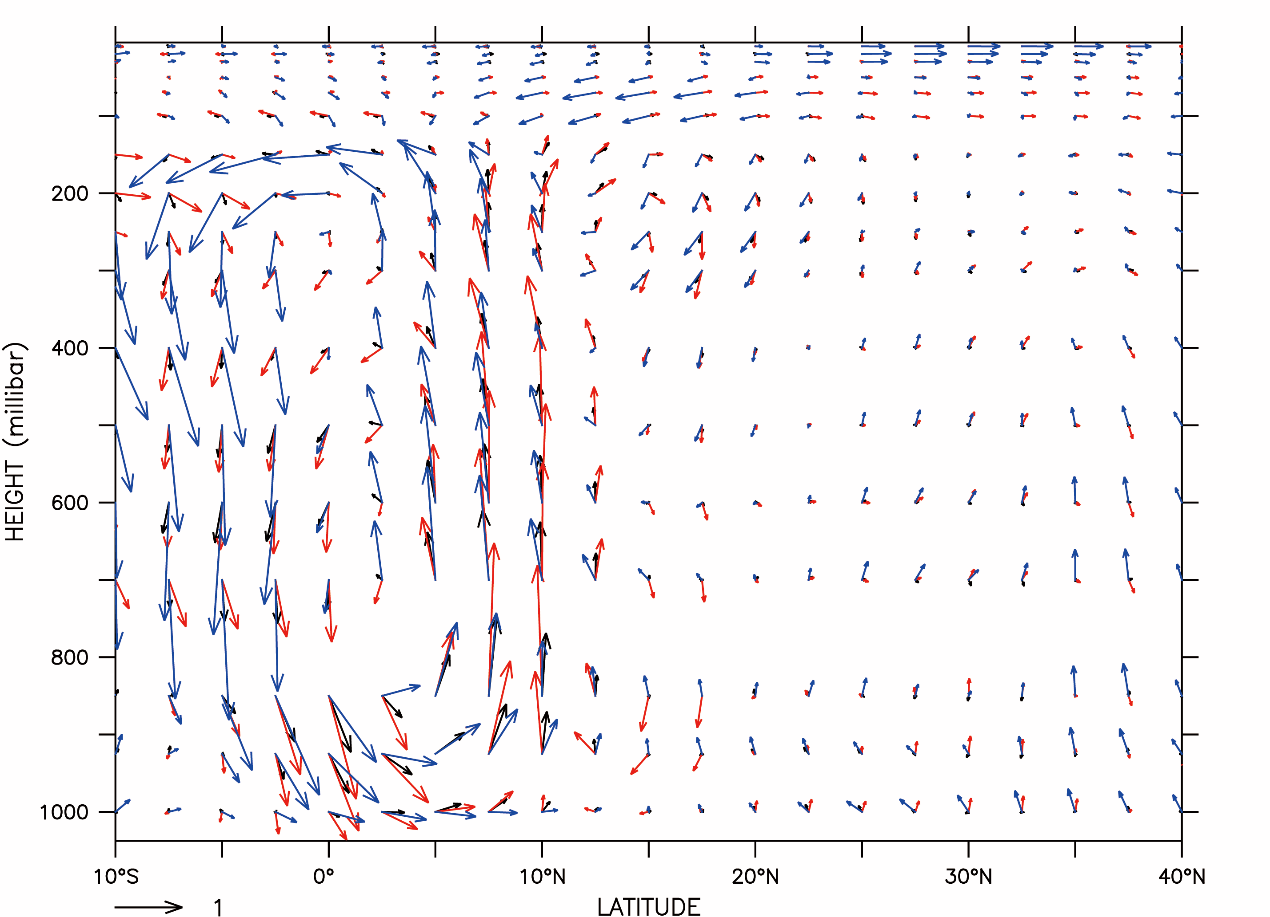


**Figure S2.** Difference of atmospheric meridional circulation between the AMO positive and negative phases during 1980-2013 based on NCEP-r2 dataset. Black, red, and blue curves indicate the global, Pacific (120°E-90°W), and Atlantic (60°W-0). The vertical velocities (unit in Pa s^-1^) are multiplied by -100.


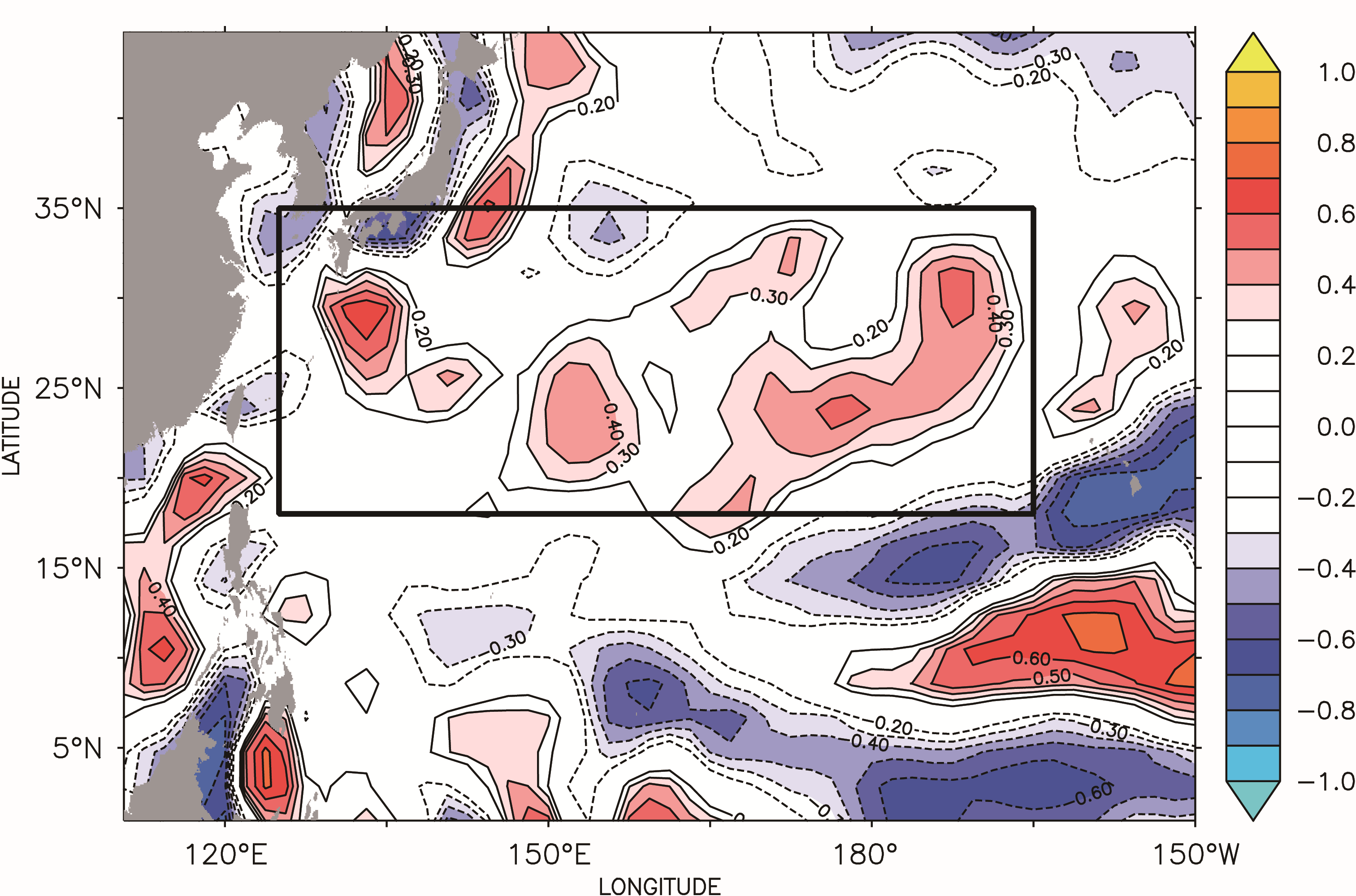


**Figure S3.** Correlation map (the AMO leads 13 months) between the AMO index and WSCA from 1980 to 2013. Colored shading indicates statistical significance above the 90% confidence level. Contour interval is 0.1.


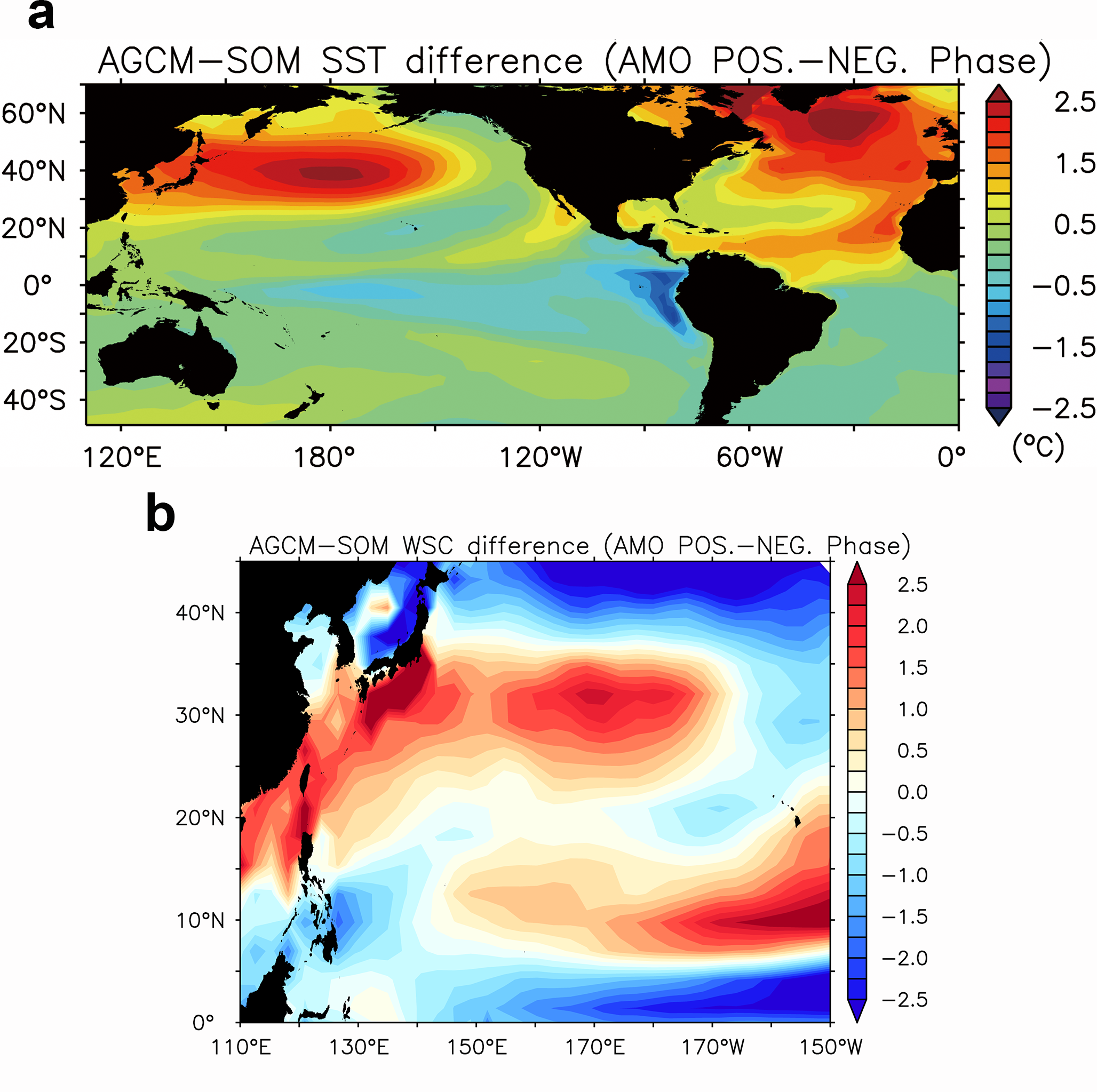


**Figure S4.** The AMO-related (a) sea surface temperature (SST, in units of °C), (b) wind stress curl (WSC, in units of 10^-8^ N m^-3^) from the AGCM–SOM experiment, calculated as differences between the AMO positive and negative runs of the model. Note that in (a) the North Atlantic SSTs were prescribed based on the regressions onto the AMO index.

**Table S1.** Correlation between indices

| **Correlation coefficient\**  **lag month** | **AMO**  **(1980-2013)** | **ITCZ**  **(1980-2013)** | **NECBL**  **(1980-2013)** |
| --- | --- | --- | --- |
| **AMO** |  | 0.38  (P=0.051) | -0.44  (P=0.020) |
| **ITCZ** | 13 (AMO leads) |  | -0.48  (P=0.005) |
| **NECBL** | 13~14 (AMO leads) | 0~1 (ITCZ leads) |  |

**Datasets**

**a. Wind speed data** are from National Centers for Environmental Prediction/Department of Energy (NCEP/DOE) AMIP Reanalysis-2 (https://www.esrl.noaa.gov/psd/data/gridded/data.ncep.reanalysis2.html) with a 2.5° latitude by 2.5° longitude resolution. The NCEP/DOE AMIP Reanalysis-2 (R-2) is an improved version of the NCEP/NCAR Reanalysis-1 (R-1) that fixed errors and updated parameterizations of physical processes.

**b. The ITCZ index** is defined as precipitation anomalies averaged over the domain between 0–10°N and 130–160°E based on GPCP data^3^.

**c. The NEC bifurcation latitude** is adopted using a wind-forced Rossby wave model (1979-1992), and calculated from monthly AVISO data (after 1992)^4^.

**d. The AMO index** is calculated as the detrended SSTA averaged over the North Atlantic from the equator to the 70°N^5^.

**e. The PDO index** is provided by the Joint Institute for the Study of the Atmosphere and Ocean (JISAO; http://research.jisao.washington.edu/pdo/).

**Statistical analyses**

Lagged correlations have been made from low-pass-filtered time series. Significance level was calculated using a standard *t*-test. Auto-correlation is taken into account by adjusting the effective number of independent observations.

**References**

1. Collins, W. D. et al. The formulation and atmospheric simulation of the Community Atmosphere Model version 3 (CAM3). *J. Clim.* 19, 2144–2161 (2006).
2. Lyu, K. & Yu, J. Y. Climate impacts of the Atlantic Multidecadal Oscillation simulated in the CMIP5 models: A re-evaluation based on a revised index, *Geophys. Res. Lett.*, 44, (2017).
3. Lin, Y. F., Wu, C. R., & Han, Y. S. A combination mode of climate variability responsible for extremely poor recruitment of the Japanese eel (Anguilla japonica). *Scientific Reports*, **7** (2017).
4. Qiu, B., & Chen, S. [Interannual-to-decadal variability in the bifurcation of the North Equatorial Current off the Philippines.](http://www.soest.hawaii.edu/oceanography/bo/QC_NEC.pdf) *J. Phys. Oceanogr.* **40**, 2525-2538 (2010).
5. Enfield, D. B., Mestas-Nunez, A. M. & Trimble, P. J. The Atlantic Multidecadal Oscillation and its relationship to rainfall and river flows in the continental US. *Geophys. Res. Lett.* **28**, 2077–2080 (2001).
